# Genotype positive hypertrophic cardiomyopathy is associated with myocardial perfusion abnormalities

**DOI:** 10.1186/1532-429X-16-S1-P342

**Published:** 2014-01-16

**Authors:** Niall G Keenan, Sneha Varkey, Rachel J Buchan, Angharad M Roberts, James S Ware, Claire E Raphael, Ankur Gulati, Dudley J Pennell, Sanjay K Prasad, Stuart A Cook

**Affiliations:** 1 Cardiology, Imperial College, London, UK; 2 Department of Cardiology, Hammersmith Hospital, London, UK; 3 CMR Unit, Royal Brompton Hospital, London, UK; 4 Royal Brompton Hospital Biomedical Research Unit, Imperial College, London, UK; 5 Department of Cardiology, National Heart Centre, Singapore, Singapore

## Background

Hypertrophic cardiomyopathy (HCM) is a highly heterogenous disease both genotypically and phenotypically. Cardiovascular magnetic resonance (CMR) with late gadolinium enhancement (LGE) for fibrosis detection and adenosine stress perfusion are key techniques for HCM phenotyping. Recent advances in DNA sequencing, in particular high throughput next generation sequencing (NGS), have enabled more extensive genetic analysis of larger cohorts of patients. We sought to assess the link between genotype and the microvascular circulation in a cohort of patients with HCM.

## Methods

We recruited 288 patients with HCM according to standard criteria, who were undergoing clinical CMR. All patients had assessment of LV volumes, mass and function and LGE, except 1 for volumes and 2 for LGE. Overall, 214 (74%) also underwent adenosine stress perfusion. Detailed demographic and outcome data were collected over a mean of 3 years follow up. 120 genes with a known or putative role in cardiomyopathy were sequenced in every patient using SureSelect target DNA capture and SOLiD next-generation sequencing, and variants were identified using GATK and LifeScope. Subjects were stratified according to the presence or absence of a likely causative genetic variant in the principal sarcomeric HCM genes.

## Results

Clinical data are presented in table 1 and genetic data in table 2. Thirty-five percent of patients carried a likely causative variant (genotype positive). Genotype positive patients were younger at diagnosis (p = 0.005), with a trend to a higher death rate during follow up. Adenosine stress perfusion was more frequently abnormal in genotype positive patients (82% v. 64%, p = 0.015). LV mass index was on average 12% greater in genotype negative patients (p = 0.005). There were no significant differences between groups for LV volumes, maximum wall thickness, or extent of LGE.

**Table 1:** Genotype Phenotype correlations in HCM [mean (SD) unless stated]

Variable	Genotype POSITIVE	Genotype NEGATIVE	Significance (NS = not significant)
Number (%)	102 (35%)	186 (65%)	

Male sex (%)	75 (73.5%)	137 (73.7%)	NS

BSA (m2)	1.97 (0.22)	1.94 (0.22)	NS

Age at CMR (years)	54.6 (12.8)	59.2 (13.4)	p = 0.005

Positive family history (%)	22 (21.6%)	35 (18.8%)	NS

Length of follow up (months)	37.6 (17.0)	40.8 (20.4)	NS

Dead (%)	6 (5.9%)	4 (2.2%)	NS

LVEDV (mL)	137.8 (33.6)	134.2 (37.2)	NS

LVESV (mL)	34.4 (16.0)	36.2 (19.4)	NS

LVSV (mL)	103.4 (25.6)	98.0 (25.0)	NS (p = 0.08)

LVEF (%)	75.5 (8.5)	74.1 (8.5)	NS

LV Mass (g)	174.8 (54.6)	191.6 (66.2)	p = 0.030

LVEDVi (mL/m2)	69.9 (14.5)	69.0 (17.0)	NS

LVESVi (mL/m2)	17.4 (7.8)	18.4 (9.2)	NS

LV mass index (g/m2)	88.1 (27.6)	98.5 (31.2)	p = 0.005

STRESS PERFUSION SCAN POSITIVE	65/79 (82.3%)	87/135 (64.4%)	p = 0.015

Max wall thickness (mm)	18.6 (3.7)	19.1 (5.1)	NS

Base most affected (%)	68 (66.7%)	103 (55.4%)	NS (p = 0.08)

Apex most affected (%)	15 (14.7%)	39 (21.0%)	NS

Septum most affected (%)	92 (90%)	154 (82.8%)	NS

LGE score (%)			

0	21 (21%)	36 (20%)	NS

1	36 (35%)	64 (35%)	NS

2	37 (36%)	68 (37%)	NS

3	8 (8%)	16 (9%)	NS

LGE score: 0=none, 1= minimal, 2= moderate, 3= widespread - as assessed by CMR level 3 accredited observers

**Table 2:** Genotype positive by gene

Gene	Number (%)
MYBPC3	31 (30%)

MYH7	26 (25%)

TNNT2	6 (6%)

Other	39 (38%)

## Conclusions

The main finding of this study is that genotype positive patients are more likely to have abnormal myocardial perfusion. It is known that genotype positive HCM patients have a higher rate of sudden cardiac death (and a trend towards death was seen in this study), and this has been postulated to be related to ischemia. Our finding that sarcomeric variants were associated with lower LV mass was unexpected and is worthy of further study given the comparatively large size of this cohort. Previous studies have highlighted the association between maximum LV wall thickness and extent of LGE with the presence of a known HCM mutation, but this finding was not replicated in the present study. More comprehensive genetic analyses enabled by next generation technologies, coupled with cutting-edge phenotypic evaluation, may enable the dissection of genotype-phenotype relationships in HCM.

## Funding

This work was supported by the National Institute of Health Research Cardiovascular Biomedical Research Unit at the Royal Brompton Hospital and Imperial College, London.

